# Serum dickkopf-1 as a clinical and prognostic factor in non-small cell lung cancer patients with bone metastases

**DOI:** 10.18632/oncotarget.18446

**Published:** 2017-06-12

**Authors:** Rong Qiao, Runbo Zhong, Qing Chang, Jiajun Teng, Jun Pei, Baohui Han, Tianqing Chu

**Affiliations:** ^1^ Department of Pulmonary Medicine, Shanghai Chest Hospital, Shanghai Jiao Tong University, Shanghai 200030, People’s Republic of China; ^2^ Shanghai Jiao Tong University School of Medicine, Shanghai 200025, People’s Republic of China

**Keywords:** dickkopf-1, non-small cell lung cancer, bone metastasis, prognosis, stage IV

## Abstract

**Background:**

The study was designed to evaluate the association between serum dickkopf-1 (DKK1) and non-small cell lung cancer (NSCLC) bone metastases.

**Materials and Methods:**

Serum DKK1 levels were quantified in 470 NSCLC patients, 140 with osseous metastases, 178 with extraosseous metastases, and 152 with early stage in complete remission. The Receiver Operating Characteristic (ROC) curve enabled us to identify a threshold value to distinguish patients with bone metastases.

**Results:**

Serum DKK1 levels in patients with osseous metastases were significantly higher than in the other 2 groups (*P* < 0.001). ROC curves showed that the optimum cutoff was 311.8 pg/ml (area under curve 0.791, 95% confidence interval 0.739–0.843, sensitivity 77.1% and specificity 71.4%). Of interest, serum DKK1 correlated with the number of bone lesions (*P* = 0.042) and associated with the poor survival in NSCLC patients with osseous metastases (*P* = 0.029).

**Conclusions:**

Our data shows that serum DKK1 can be used for the detection of NSCLC bone metastases. More importantly this is the first report to show that serum DKK1 is a good predictor of poor prognosis in NSCLC patients with bone metastases.

## INTRODUCTION

Lung cancer is the leading cause of cancer-related mortality worldwide [1]. Late diagnosis is common, and the majority of patients present with metastatic disease. Bone is one of the predominant metastatic sites of lung cancer, accounting for 30%–40% of patients [2]. The presence of bone metastases is a poor prognostic factor that may induce clinical symptoms, such as pain, pathological fractures, paralysis and hypercalcemia, all of which adversely affect quality of life and treatment outcomes. Monitoring bone metastases is essential for lung cancer management.

Serum carcinoembryonic antigen (CEA) is considered a poor prognostic factor in early-stage non-small cell lung cancer (NSCLC) [3–13], but its role in advanced disease is controversial. In a study from Cedrés et al. (2011)[14] which included 277 advanced patients, high CEA levels at diagnosis were an unfavorable prognostic factor; however, these results were not replicated in studies from Kulpa et al. (2002) [15] and Ardizzoni et al. (2006) [16]. Therefore, novel and reliable prognostic biomarkers to complement CEA are urgently needed for clinical use in advanced NSCLC.

The Wnt/β-catenin pathway plays a crucial role in tumor pathogenesis, specifically in cell proliferation, angiogenesis, motility and invasiveness [17–19], and may also play an important role in the differentiation of osteoblasts [20, 21]. The activity of Wnt family ligands is antagonized by several secreted factors including Dickkopf (DKK). DKK1 blocks Wnt/β-catenin signaling by binding low-density lipoprotein receptor-related protein 5 and 6 and preventing receptor-ligand interactions [22, 23]. It has been reported that DKK1 produced by myeloma cells inhibits osteoblast differentiation *in vitro*, and increased DKK1 levels in bone marrow plasma and peripheral blood correlate with the presence of lytic bone lesions in myeloma patients [20]. It has also been suggested that breast cancer cells with over-activated Wnt/β-catenin signaling produce high levels of DKK1, which is involved in breast cancer-derived osteolytic metastases [24]. As our previous studies described, we have found that lung tumor cell derived DKK1 increases the level of β-catenin and negatively regulates osteoblast differentiation [25], indicating that lung cancer produced DKK1 may be an important mechanistic link between NSCLC and bone metastases.

However, whether serum DKK1 contributes to bone metastasis and can be used to predict the prognosis of advanced NSCLC patients has not been addressed. In this study, we investigated whether circulating DKK1 levels were associated with bone metastases in NSCLC and explored additional information relating to the severity and prognosis of NSCLC.

## RESULTS

### Serum DKK1 levels are increased in NSCLC patents with bone metastases

This study enrolled 470 NSCLC patients, including 140 with osseous metastases, 178 with extraosseous metastases, and 152 with early stage in complete remission, and DKK1 protein was detected in all serum samples. The baseline characteristics of all patients are shown in Table 1. The mean level of serum DKK1 was 371.44 pg/ml in patients with osseous metastases. In contrast, the mean levels of serum DKK1 were 260.71 pg/ml and 160.07 pg/ml in patients with extraosseous metastases and patients with early stage in complete remission, respectively. The expression of DKK1 in serum was significantly upregulated in patients with osseous metastases compared to patients with early stage in complete remission (*P* < 0.001) and patients with extraosseous metastases (*P* < 0.001). In stage IV, as shown in Table 2, serum DKK1 did not correlate with *N* stage (*P* = 0.455), *T* stage (*P* = 0.761), pleura metastases (*P* = 0.472), lung metastases (*P* = 0.928), brain metastases (*P* = 0.186), liver metastases (*P* = 0.800) or adrenal gland metastases (*P* = 0.838). Using the Receiver Operating Characteristic(ROC) curve drawn with these data, a serum level of 311.8 pg/ml was selected as the optimal cutoff value for detecting NSCLC with bone metastases to get both high sensitivity and high specificity (area under curve [AUC] 0.791, 95% confidence interval [CI] 0.739–0.843, sensitivity 77.1% and specificity 71.4%). These findings indicated that DKK1 is a potential serum biomarker to distinguish between osseous metastases and extraosseous metastases in stage IV NSCLC patients.

**Table 1:** Clinical features of all patients

Characteristics	patients with early stage (*N* = 152)	Patients with osseous metastases (*N* = 140)	Patients with extraosseous metastases (*N* = 178)
**Mean age (years)**	59.51	57.1	59.1
**Gender**			
male	98	87	111
female	54	53	67
**ECOG PS**			
0–1	150	134	174
2	2	6	4
**Smoking history**			
yes	89	54	93
no	63	86	85
**Histology**			
adenocarcinoma	96	100	120
squamous carcinoma	32	15	22
other NSCLC	24	25	36

Abbreviations: NSCLC, non-small cell lung cancer; ECOG PS, Eastern Cooperative Oncology Group performance status; *N*, number.

**Table 2:** Serum DKK1 concentrations in different metastatic organs in all stage IV NSCLC patients (*N* = 318)

Variables	Subgroup	*N*	DKK1 pg/ml(mean)	*P* value
**Bone metastases**				< 0.001
	yes	140	371.44	
	no	178	260.71	
**Pleura metastases**				0.472
	yes	135	303.74	
	no	183	313.68	
**Lung metastases**				0.928
	yes	99	310.39	
	no	219	309.04	
**Brain metastases**				0.186
	yes	79	324.68	
	no	239	304.43	
**Liver metastases**				0.800
	yes	29	315.66	
	no	289	308.84	
**adrenal gland metastases**				0.838
	yes	19	315.61	
	no	299	309.07	
**N stage**				0.455
	N0 + N1	49	299.39	
	N2 + N3	269	311.29	
**T stage**				0.761
	T1 + T2	177	311.27	
	T3 + T4	141	307.20	

Abbreviations: NSCLC, non-small cell lung cancer; DKK1, Dickkopf-1; N, number.

**Figure 1:**
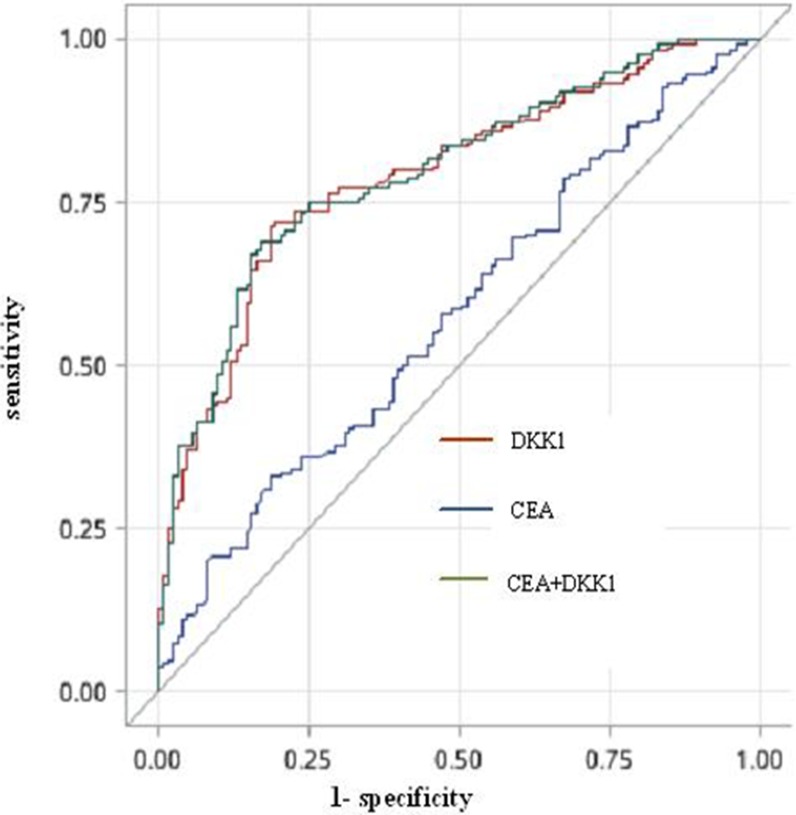
The ROC curve for DKK1, CEA or both for NSCLC patients with osseous metastases versus the extraosseous metastases Abbreviations: DKK1, Dickkopf-1; CEA, carcinoembryonic antigen; ROC, receiver operating characteristic.

To evaluate the clinical utility of serum DKK1 as a bone metastasis biomarker, we also measured the conventional tumor marker CEA in patients with stage IV. Serum CEA levels were abnormally elevated in 180 out of 287 (62.7%) patients, and significantly higher in the osseous metastases group than the extraosseous metastases group (*P* = 0.042). The optimum cutoff value for CEA was 3.03 ng/ml (AUC 0.577, 95% CI 0.511–0.644, sensitivity 81.3% and specificity 67.1%). ROC analysis showed that testing both DKK1 and CEA increased the detection accuracy for NSCLC bone metastases compared with CEA alone (AUC 0.797, 95% CI 0.746–0.848, sensitivity 82.9% and specificity 68.9%; DKK1 plus CEA vs. DKK1 alone *P* = 0.370; DKK1 plus CEA vs. CEA alone *P* = 0.0001). Thus, serum DKK1 had a greater AUC than CEA in patients with osseous metastases compared with patients with extraosseous metastases (Figure 1, Table 3). As determined by bivariate correlation analysis, the correlation coefficient between serum DKK1 and CEA levels was not significant (correlation coefficient 0.049; *P* = 0.407), also indicating that measuring both serum markers could improve the overall sensitivity for detecting bone metastases.

**Table 3:** Results for measurement of serum DKK1, CEA or both in detection of bone metastases

Variables	AUC (95% CI)	Cutoff value	Sensitivity (%)	Specificity (%)
**CEA**	0.577	3.03 (ng/ml)	81.3	67.1
	(0.511–0.644)			
**DKK1**	0.791	311.8 (pg/ml)	77.1	71.4
	(0.739–0.843)			
**CEA + DKK1**	0.797		82.9	68.9
	(0.746–0.848)			

Abbreviations: DKK1, Dickkopf-1; CEA, carcinoembryonic antigen; ROC, receiver operating characteristic; AUC, area under curve.

### Association between serum DKK1 levels and clinicopathological parameters in patients with osseous metastases

In the osseous metastases group, 45 patients had a solitary bone metastasis and 95 had multiple bone metastases; 49 patients had isolated bone metastases and 91 had bone and visceral or cerebral metastases. Serum DKK1 levels were classified as high or low in relation to the cutoff value, which was based on the results of ROC analysis. Patients with osseous metastases (*n* = 140) were divided into groups based on serum DKK1 levels, the high level group (DKK1 > 311.8 pg/ml, *n* = 108) and a low level group (DKK1 ≤ 311.8 pg/ml, *n* = 32). The relationship between serum DKK1 level and clinicopathological characteristics of these patients were then analyzed as shown in Table 4. High serum DKK1 levels were significantly correlated with age (*P* = 0.017), and intriguingly, also with the number of bone metastases (*P* = 0.042). There was no association with metastatic pattern and the distribution of bone metastases.

**Table 4:** Association between serum DKK1 and clinicopathological characteristics of NSCLC patients with bone metastases (*N* = 140)

Variables	DKK1		*P* value
	Low	High	
**Mean age (years)**			0.017
≤ 57.1	21	45	
> 57.1	11	63	
**Gender**			0.644
male	21	66	
female	11	42	
**ECOG PS**			0.351
0–1	32	101	
2	0	7	
**Smoking history**			0.579
yes	11	43	
no	21	65	
**Histology**			0.077
adenocarcinoma	27	73	
nonadenocarcinoma	5	35	
**N stage**			0.330
N0 + N1	6	13	
N2 + N3	26	95	
**T stage**			0.810
T1 + T2	20	70	
T3 + T4	12	38	
**Number of bone metastases**			0.042
solitary bone metastases	15	30	
multiple bone metastases	17	78	
**Metastatic pattern**			0.353
additional visceral or cerebral metastases	23	68	
isolated bone metastases	9	40	
**Distribution of bone metastases**			
thorax			0.643
yes	19	69	
no	13	39	
vertebrae			0.317
yes	17	68	
no	15	40	
pelvis			0.317
yes	15	40	
no	17	68	
limb			0.135
yes	14	32	
no	18	76	
skull			0.987
yes	5	17	
no	27	91	

Abbreviations: ECOG PS, Eastern Cooperative Oncology Group performance status; DKK1, Dickkopf-1.

### High serum DKK1 levels are associated with shorter overall survival in patients with osseous metastases

**Figure 2:**
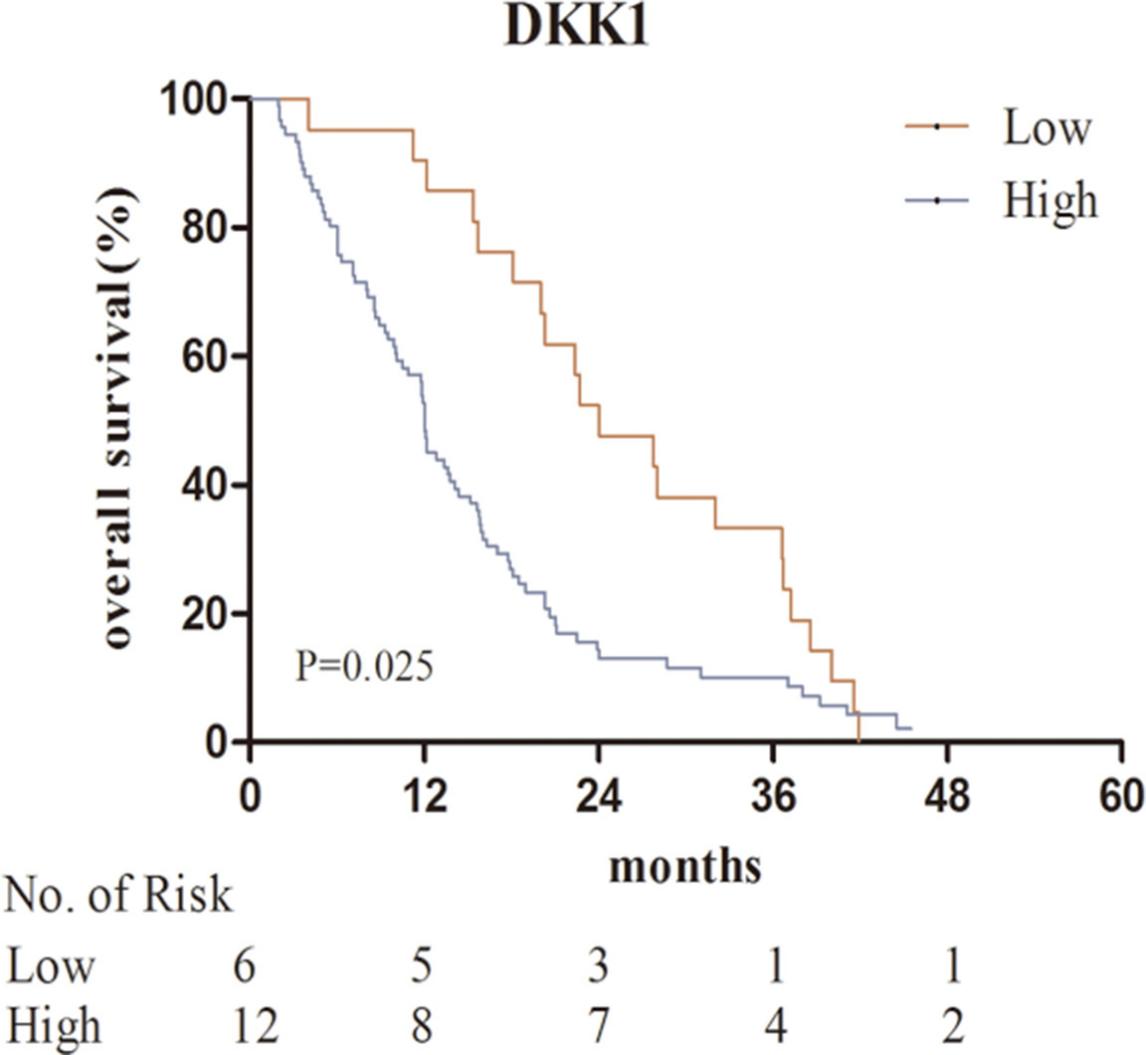
Kaplan–Meier analysis for serum DKK1 in the bone metastases group Abbreviations: DKK1, Dickkopf-1.

**Figure 3:**
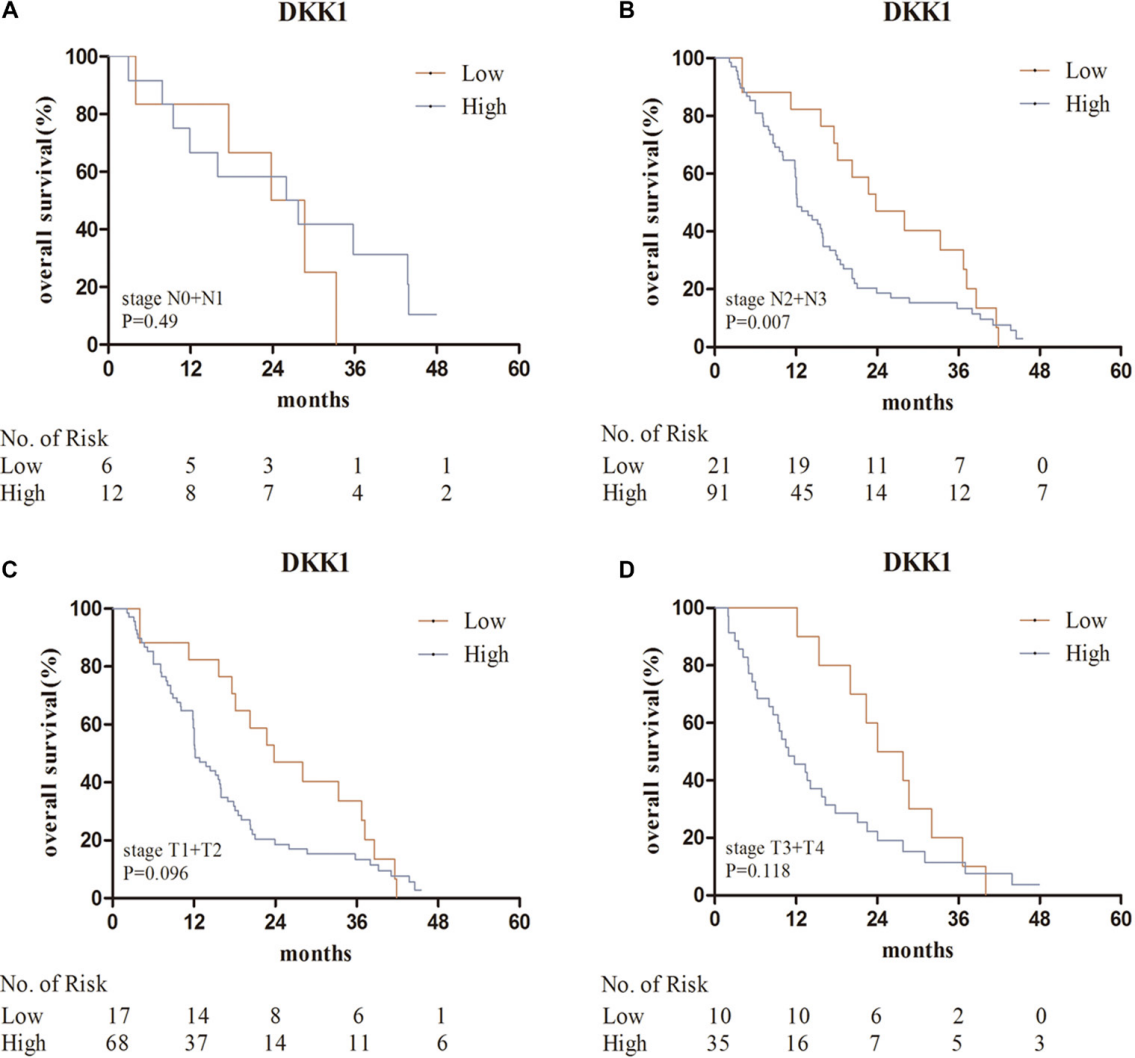
Kaplan–Meier analysis for serum DKK1 in the subgroups of bone metastases (**A**, **B**) patients with stage N0 + N1, N2 + N3; (**C**, **D**) patients with stage T1 + T2, T3 + T4. Abbreviations: DKK1, Dickkopf-1.

**Figure 4:**
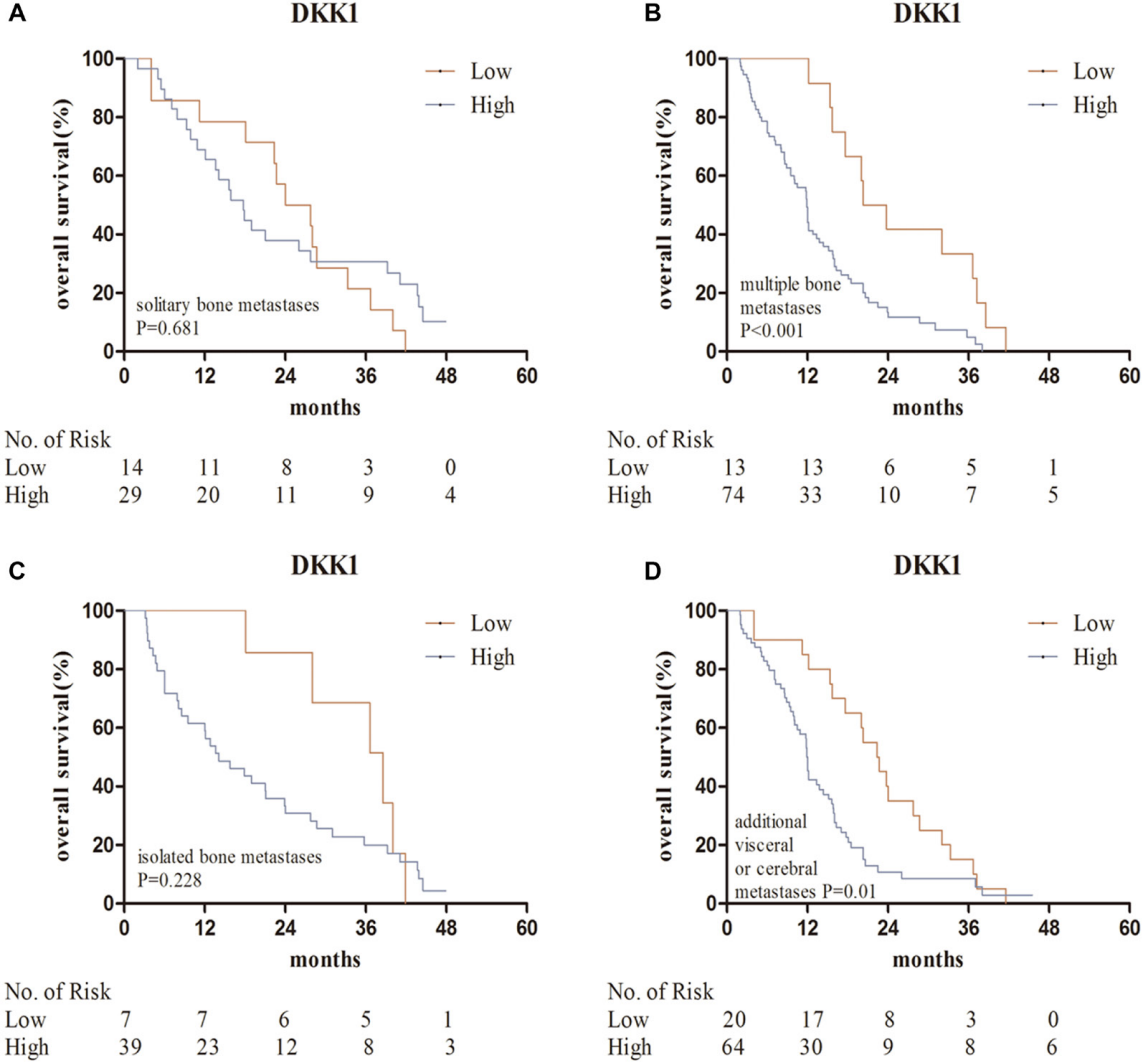
Kaplan–Meier analysis for serum DKK1 in the subgroups of bone metastases (**A**, **B**) patients with solitary bone metastasis, multiple bone metastasis; (**C**, **D**) patients with isolated bone metastases, bone and visceral or cerebral metastases. Abbreviations: DKK1, Dickkopf-1.

The median survival time of patents with bone metastases was 15.2 months. Kaplan–Meier analysis indicated that high DKK1 levels were correlated with poor overall survival (*P* = 0.025) (Figure 2). Predictive value of serum DKK1 in the subgroups of bone metastases, such as in those with solitary bone metastasis, multiple bone metastasis, isolated bone metastases, additional visceral or cerebral metastases, stage N0 + N1, stage N2 + N3, stage T1 + T2 and stage T3 + T4 were further investigated. Serum DKK1 also showed prognostic significance in the subgroups of patients with stage N2 + N3, multiple bone metastasis, and additional visceral or cerebral metastases (Figures 3, 4).

Univariate Cox regression analyses revealed significant associations between overall survival and ECOG PS (hazard ratio [HR] = 3.115; *P* = 0.004), *N* stage (HR = 1.862; *P* = 0.026), number of bone metastases (HR = 2.088; *P* = 0.001), metastatic pattern (HR = 1.297; *P* = 0.009) and serum DKK1 level (HR = 1.648; *P* = 0.026). There were no associations between OS and age, sex or smoking history. Multivariate Cox regression analysis indicated that ECOG PS (HR = 3.956; *P* = 0.001), serum DKK1 level (HR = 1.675; *P* = 0.029), number of bone metastases (HR = 1.904; *P* = 0.004) and metastatic pattern (HR = 1.283; *P* = 0.022) were independent prognostic factors for overall survival in NSCLC patients with bone metastases (Table 5).

**Table 5:** Univariate and multivariate analyses for prognostic factors in NSCLC patients with bone metastases (*N* = 140)

Variables	OS (univariate)	*P* value	OS (multivariate)	*P* value
	Hazard ratio (95% CI)		Hazard ratio (95% CI)	
**Age**	1.036	0.850	1.124	0.554
(≤ 57.1vs. > 57.1)	(0.722–1.486)		(0.763–1.655)	
**Gender**	0.970	0.870	0.657	0.095
(male vs. female)	(0.671–1.402)		(0.401–1.076)	
**ECOG PS**	3.115	0.004	4.053	0.001
(0–1 vs. 2)	(1.429–6.790)		(1.799–9.127)	
**Smoking history**	0.937	0.730	0.679	0.131
(yes vs. no)	(0.647–1.357)		(0.411–1.122)	
**N phase**	1.862	0.026	1.565	0.132
(N0+N1 vs. N2+N3)	(1.077–3.217)		(0.874–2.802)	
**Number of bone metastases**	2.088	0.001	1.904	0.004
(solitary vs. multiple)	(1.379–3.161)		(1.227–2.954)	
**Metastatic pattern**	1.297	0.009	1.283	0.022
(isolated bone vs. bone and other organs)	(1.066–1.578)		(1.036–1.588)	
**Serum DKK1 levels**	1.648	0.026	1.675	0.029
(low vs. high)	(1.060–2.561)		(1.055–2.660)	

Abbreviations: NSCLC, non-small cell lung cancer; OS, overall survival; ECOG PS, Eastern Cooperative Oncology Group performance status; CI, confidence interval; DKK1, Dickkopf-1.

## DISCUSSION

DKK1 is a potent inhibitor of Wnt/β-catenin signaling [18, 26, 27] and has also been shown to inhibit osteoblastogenesis in various osteogenic cell lines [20, 28–31]. Elevated serum DKK1 has been found to be associated with worse survival in cancers from multiple organs including the pancreas [32], stomach [33, 34], liver [35, 36], ovary [37], bile duct [38], bladder [39, 40], prostate [41, 42], breast [43, 44], cervix [45, 46] and esophagus [47, 48]. Meanwhile, several studies investigated the clinical and prognostic significance of serum DKK1 in NSCLC, and concluded that DKK1 levels increased with the presence of distant metastases, and correlated with poor overall survival [49–51]. Despite these studies, the involvement of DKK1 in lung cancer bone metastases was still unclear. In this study, we found that serum DKK1 levels were significantly higher in patients with bone metastases compared with patients with early stage in complete remission (*P* < 0.001) and patients with extraosseous metastases (*P* < 0.001). Using ROC curves drawn from our results, we obtained a sensitivity of 77.1% and a specificity of 71.4% for detecting NSCLC with bone metastases with a DKK1 cutoff level of 311.8 pg/ml. Combining serum DKK1 and CEA increased the sensitivity to 82.9%, significantly higher than CEA alone (*P* = 0.0001), and there was no correlation between CEA and DKK1. No statistically significant differences between serum DKK1 levels and different extraosseous metastatic sites (pleura, lung, brain, liver and adrenal gland) were found. The reason why serum DKK1 was slightly lower in pleura metastases group in comparison with non-pleura metastases group may be due to the limited number of patient samples. Furthermore, we found that patients with higher serum DKK1 levels were more likely to have multiple bone metastases. However, we also found that serum DKK1 levels did not differ significantly with metastatic pattern and the distribution of bone metastases. Our study suggested that elevated serum DKK1 level could be a marker for the presence of bone metastases in NSCLC, as well as breast cancer [43], multiple myeloma [52] and prostate cancer [53].

With respect to the results of survival analysis in patients with bone metastases, multivariate analysis identified that serum DKK1 levels and number of bone metastases were independent prognostic predictors for OS (*P* = 0.029, *P* = 0.004, respectively). Moreover, Kaplan–Meier analysis indicated that high serum DKK1 levels were correlated with poor overall survival (*P* = 0.025). Further prognostic analysis in clinical subgroups showed high serum DKK1 levels were associated with bad survival in patients with multiple bone metastasis. All these results confirmed that serum DKK1 could be used to predict the prognosis of NSCLC patients with bone metastases. To our knowledge, this is the first study to comprehensively investigate the association between serum DKK1 and NSCLC bone metastases. Because the number of serum samples we tested was limited, our results are just preliminary and might have some limits. A larger number of lung cancer samples need to be collected and more studies are also needed to corroborate our results. Further investigation on the function and mechanism of DKK1 in the bone metastasis of NSCLC is awaited.

Our results suggested that DKK1 plays an important role in lung cancer bone metastasis, and serum DKK1 may be a potential tumor biomarker of clinical prognostic value. No reported studies have investigated serum DKK1 in NSCLC patients with bone metastases. However, the mechanism underlying why DKK1 overexpression predicted poor prognosis for NSCLC patients with bone metastases is unclear. Further studies are required to delineate the role of DKK1 in the bone metastases of NSCLC.

## MATERIALS AND METHODS

### Patients and serum samples

Serum samples were collected from 140 patients with osseous metastases, 178 patients with extraosseous metastases, and 152 patients with early stage in complete remission admitted to Shanghai Chest Hospital from October 2011 to April 2012. Blood samples were collected before any treatment, and stored at −80°C. The study was approved by the ethical committees of Shanghai Chest Hospital and informed consent was signed by each patient. The enrollment criteria for all patients in this study were as follows: 1) pathologically proven primary NSCLC diagnosis; 2) Eastern Cooperative Oncology Group performance status (ECOG PS) 0–2; 3) life expectancy not less than 3 months; 4) no history of other cancers.

The following factors were evaluated: age; sex; histology; extent of primary lesion (*T* stage); involvement of regional lymph nodes (*N* stage); evidence of metastases to brain, liver, bone, lung, pleura, lymph node, pericardium, subcutaneous nodule or adrenal gland (M stage); performance status grade; serum DKK1 and CEA levels. Tumor stage was determined according to the 2009 tumor-node-metastasis (TNM) staging system.

Serum DKK1 levels were measured by enzyme-linked immunosorbent assay (ELISA) with an immunoassay kit (Miltenyi, Bergisch Gladbach, Germany) according to the manufacturer’s directions and measuring optical density at 450 nm. Results are reported as the concentration of DKK1 (pg/ml) in samples. Serum CEA levels were analyzed using a Quantitative assay kit (Roche Diagnostic GmbH, Basel, Switzerland) with an upper limit of 5 ng/ml defined as normal according to the kit’s manufacturer.

Skeletal metastases were classified into five regions [54]: thorax (rib, clavicle, sternum and scapula), vertebrae (thoracic, lumbar and cervical spine), pelvis (ilium, ischium, pubis, sacrum and sacroiliac regions), limb (upper and lower extremities) or skull. Metastatic load was divided into solitary and multiple bone metastases. Metastatic pattern was divided into isolated bone and bone with visceral or cerebral metastases.

### Statistical analysis

Overall survival (OS) was calculated from the date of diagnosis until the date of mortality or the last follow-up date. Differences in serum DKK1 levels between groups were assessed by *t* test. Correlations between serum DKK1 levels and clinicopathological characteristics were estimated using *χ*^2^ or Fisher’s exact test. Receiver operating characteristics (ROC) curves were constructed to assess sensitivity, specificity and respective areas under the curves (AUCs) with 95% confidence intervals (CIs). We investigated the optimum cutoff value by maximizing the sum of sensitivity and specificity. Survival curves were estimated using the Kaplan–Meier method, and the log-rank test was used to compute differences between the curves. Multivariate analysis using the Cox proportional hazards regression model was performed to assess the prognostic value of serum DKK1 levels. All *P* values were two-tailed, *P* < 0.05 was taken to be significant and 95% CIs for results were calculated, where appropriate. Data were analyzed using SAS 9.4 (SAS Institute, Cary, NC, USA).
